# A Mobile Crowd Sensing Application for Hypertensive Patients

**DOI:** 10.3390/s19020400

**Published:** 2019-01-19

**Authors:** Slađana Jovanović, Milan Jovanović, Tamara Škorić, Stevan Jokić, Branislav Milovanović, Konstantinos Katzis, Dragana Bajić

**Affiliations:** 1 Telekom Srbija A.D. Takovska 2, Belgrade 11000, Serbia; sladjanajo@telekom.rs; 2 Endava, Bulevar Milutina Milankovića 11, Belgrade 11000, Serbia; milan.jovanovic@endava.com; 3 Faculty of Technical Sciences, University of Novi Sad, Trg. D. Obradovića 6, Novi Sad 21000, Serbia; ceranic@uns.ac.rs; 4 Svezdrav Rešenja LLC, Đenerala Draže 44, Klenje 15357, Serbia; stevan@ecg4everybody.com; 5 Faculty of Medicine, University of Belgrade, Dr Subotića 8, Belgrade 11000, Serbia; branislav_milovanovic@vektor.net; 6 Department of Computer Science and Engineering, European University Cyprus, Diogenis Str 6, Nicosia 1516, Cyprus; k.katzis@euc.ac.cy

**Keywords:** mobile crowd sensing, Internet of Everything, hypertension, quality of information, machine learning

## Abstract

Mobile crowd sensing (MCS) is an application that collects data from a network of conscientious volunteers and implements it for the common or personal benefit. This contribution proposes an implementation that collects the data from hypertensive patients, thus creating an experimental database using the cloud service Platform as a Service (PaaS). The challenge is to perform the analysis without the main diagnostic feature for hypertension—the blood pressure. The other problems consider the data reliability in an environment full of artifacts and with limited bandwidth and battery resources. In order to motivate the MCS volunteers, a feedback about the patient’s current status is created, provided by the means of machine-learning (ML) techniques. Two techniques are investigated and the Random Forest algorithm yielded the best results. The proposed platform, with slight modifications, can be adapted to the patients with other cardiovascular problems.

## 1. Introduction

Crowd sensing is a concept raised by the fact that nowadays the majority of the human population owns smart, wearable, mobile devices equipped with diverse sensors and able to record, process and transmit a great variety of data. These data can be stored, shared and analyzed to improve the knowledge about the phenomena of common and personal benefit, thus increasing numerous aspects of general well-being. This represents the essence of crowd sensing.

Initial crowd sensing typology included environmental, infrastructural and social applications [1]. The health issues were brought into the focus later, following the development of the necessary prerequisites considering the reliability, security, networking, communication and location accuracy [2,3,4,5]. A taxonomy of mechanisms for health crowd sensing, with an overview of the specific nature of incentivization for the case of health-related data, was presented in [6]. Case studies of particular diseases were presented in [7] and [8], while a MCS architecture in healthcare was described in [9]. The most exemplary application is DietSense that, combining social and medical aspects, supports its participants in dealing with weight problems [1].

The mobile crowd sensing in health connects things (devices and objects), people, data (converting data into intelligence to make better decisions) and processes (delivering the right information to the right person or right machine at the right time), thus aligning with the worldwide universal concept of the Internet of Everything (IoE) [10,11].

The most recent contributions, however, deal with a general theoretical approach to the crowd sensing platform. In a multivariate surrounding, the optimization is performed considering the incentivization, protection and quality of information. An extensive theoretical approaches using the most sophisticated mathematical tools [12,13] were followed by a realization of a new MCS framework [14] that integrates several complex crowd sensing mechanisms. Such schemes provide an opportunity to apply MCS in an optimal way in complex multivariate surroundings.

The aim of this paper is to employ a MCS application coupled with advanced data analysis designed for hypertensive patients in order to gain a better understanding of this particular medical condition. Hypertension is known as “the silent killer” as it exhibits no obvious symptoms; it develops slowly over the time and it can be related to many causes. The general characteristic is that the patients behave as if they were healthy, occasionally even avoiding the therapy [15]. The application purpose is to create a huge database of cardiovascular signal features. The database creation is motivated by the quantity of acquired signals that patients observe at daily bases as a routine check-up and delete straightforwardly upon the observation. The MCS concept encourages the patients to share the data for further analysis, simultaneously providing a feedback considering their current status.

The major diagnostic markers for hypertension are systolic and diastolic blood pressure (BP). The reliable BP monitoring is semi-invasive and it involves a periodic inflation/deflation of wrist (arm) cuff, or, in the Portapres^®^ device [16], a constant inflation of alternating finger cuffs. Such a monitoring is not compatible with crowd sensing. This paper creates a model to determine the patient’s status without BP as the most distinctive feature, thus making a crowd sensing application available for hypertensive patients. The features implemented in ML techniques are derived from the electrocardiogram. The paper also shows that the automatic signal correction ensures the features are of sufficient quality, despite the fact that in the MCS environment subjects are moving and sensors are not attached by medical professionals, and although in medical experiments manual artifact correction is required, this is impossible in crowd sensing. The white space of the TV spectrum is discussed as a possible candidate for an unoccupied bandwidth solution. The impact of the cardiovascular parameter that is designed specifically for crowd sensing–the binarized entropy [17]–is shown to be beneficial. Finally, the results of an already developed and spread small Android application are presented. With slight software modifications the application could easily be adapted to a different cardiovascular problem.

## 2. Materials and Methods

### 2.1. Data—Quality of Information

The amount of signals recorded using smart mobile devices can be tremendously large, as hypertensive patients do not avoid activities like fitness or exercise. Collected signals, provided that the quality of information (QoI) is assured [6], can be a valuable source of data for diagnostic and prognostic purposes. The QoI relies on correct measurement techniques and accurate data reporting. In cardiovascular MCS, QoI is directly related to three major parameters–bandwidth, battery, artifacts–abbreviated as “BBA”.

MCS implies that the processing operations are constrained by the battery, with the transmitter as the major energy consumer. The transmission data rate of a single patient might be considered as negligible, but the total number of patients and the permanency of recording accumulate a considerable load on the available bandwidth. Thus, the transmission presents a double burden, both for the bandwidth and for the battery.

**Figure 1:**
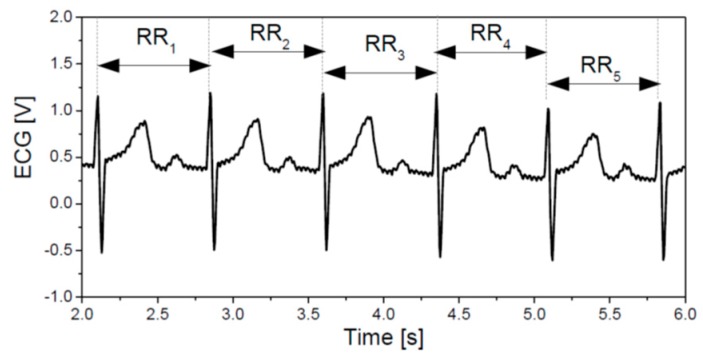
ECG signal showing six heart-beats and six intervals between the successive R peaks (RR intervals); in this Figure RR and NN intervals are identical as there are no artifacts.

The battery/bandwidth versus processor consumption trade-off was evaluated in [3], yielding a recommendation that actually become an official guideline for MCS: acquired data should be processed within the wearable smart device, and only the results should be transmitted to the remote end. A simple example shows the effects of the local analysis: ten minutes–600 s–of ECG recording, sampling frequency 1 kHz and 12 bits per sample yields 7,200,000 bits for transmission, while a single parameter extracted from ECG requires 16 or 32 bits only [13]. This leads to the third, and the most important problem: “A” stands for “artifacts”, a term generally used in medicine to describe any signal disturbance. Mobile monitoring of cardiovascular signals usually implies electrocardiogram/pulse recording. Figure 1 presents a sample of electrocardiogram (ECG) and the corresponding RR intervals—the intervals between the successive peaks of ventricular contraction (R peaks). An inverse of RR interval is instantaneous heart rate (HR) expressed in beats per minute. When all the artifacts and pathologies are removed from a series of RR intervals, these intervals become known as NN intervals and only then can be used for further processing.

The signal in Figure 1 is an idealized version. The real signals may be distorted, and the majority of artifacts are a consequence of unprofessionally and possibly loosely attached sensors. In contrast to the patients in healthcare with sensors placed by trained medical staff, MCS subjects are freely moving, thus increasing the sensor-skin friction. Another source of artifacts is the software for heart rate (RR interval) detection.

**Figure 2:**
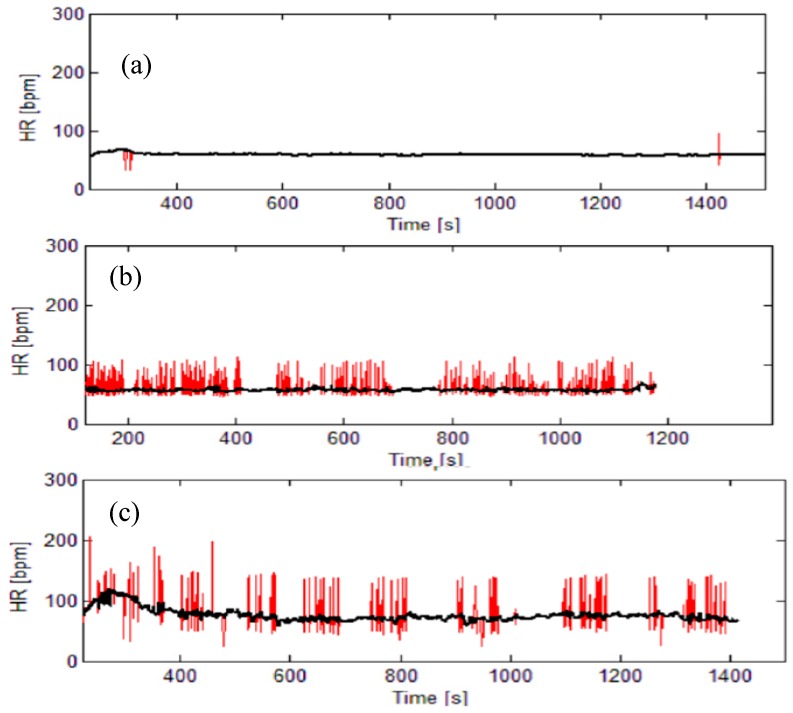
Typical heart rate (HR) signals: red lines-raw signals with artifacts, black lines—corrected signals. (**a**) almost ideal signal; (**b**) signal with artifacts; (**c**) signal with ectopic beats (and artifacts). The signals (**b**) and (**c**) cannot be used for further processing.

The signal pathologies (e.g., arrhythmia) are also considered as artifacts. Figure 2 presents an illustrative example of real heart-rate signals distorted by artifacts. Artifacts can prevent reliable signal analysis and the requirements that cardiovascular data have to fulfill in order to be further processed are strict. Most of the signal processing features explicitly require artifact-and pathology-free signals; some analysis (e.g., spectral and entropy) require stationary signals which is inconsistent with moving subjects; some of the processing tools require equidistant interpolation.

The recommendations for cardiovascular data processing [18] state that the results would be reliable if all the signal samples were of the same length, with minimal signal duration of 300 s. Besides, the ECG sampling frequency should be the same, a requirement that cannot be fulfilled if different devices are used; this issue influences the resolution of the RR intervals.

In the scientific experiments, artifacts are removed by visual inspection and each study contains a statement “after a long and tedious visual observation, the artifacts are corrected”. It is of the uttermost importance, as most of the features used for cardiovascular analysis are extracted from RR (or HR) time series. Though reliable, visual methods cannot be applied in crowd sensing where it is not expected that the subject would spend more time than necessary to turn the application on and off. To eliminate the artifacts and other obstacles in MCS applications, raw RR signals (corresponding to the red lines in Figure 2), must be pre-processed. The artifacts should be removed using a filter designed specifically for RR time series [19], while the signal stationarity should be assured by removing the slow-varying trend using another filter, also designed for RR time series [20]. If necessary, the signals should be resampled to be equidistant (1 Hz). We have avoided other interval time series that can be derived from ECG, as the software artifacts are more frequent and the algorithms for their elimination less reliable. After the pre-processing, short signals [18] and signals with too many artifacts are discarded. The remaining signals are then processed in order to extract the features which are transmitted to the remote end (cloud) and, as a feedback, an information of the subject’s status is reported.

**Figure 3:**
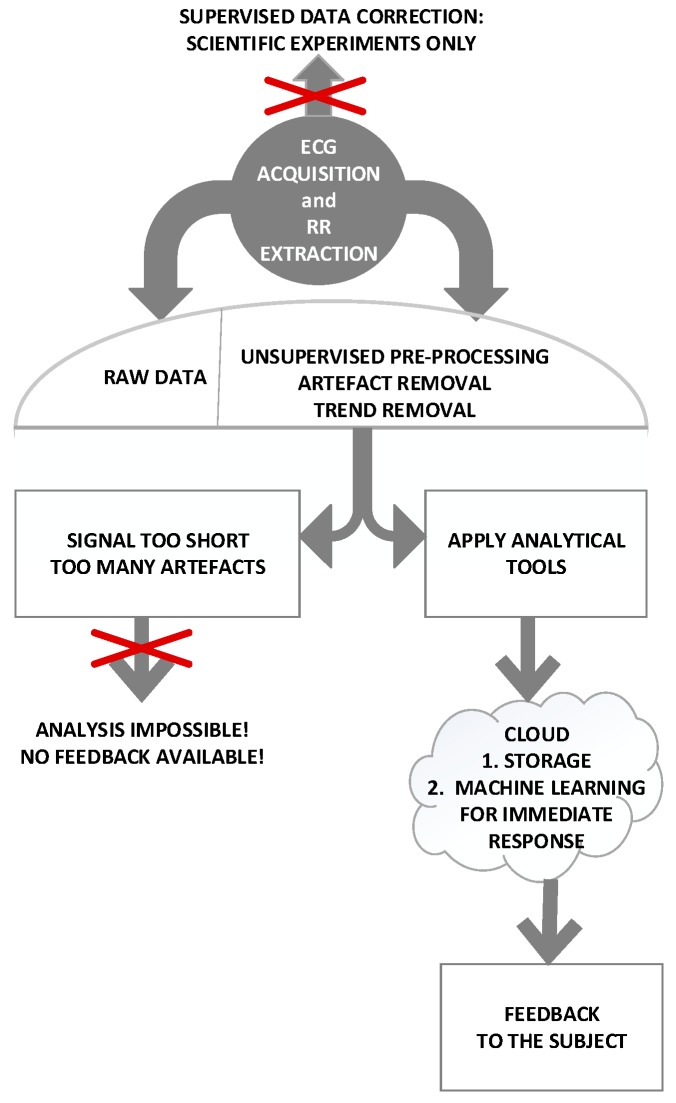
Flow diagram of the procedure. After the ECG recording and RR extraction, artefacts can be removed by visual inspection (scientific studies), or the artefact removal can be automatic (MCS). Signals that are too short or with too many artefacts are discarded. Remaining signals are analyzed and the corresponding features are transmitted to be stored in the cloud. A feedback with the subject’s status is then returned.

The flow diagram of the procedure is shown in Figure 3

### 2.2. People—Minimal Effort Principle

Crowd sensing is a volunteer action, intended for conscious subjects that wish to improve the common well-being. The purpose of this action is to form a large database considering hypertensive patients in different environmental circumstances. The task for volunteers is to get and install the application, eventually get some additional customized hardware, and start the application when they feel this is appropriate, choosing the corresponding activity—walking, working, mountain climbing, cycling, etc. The automatic activity recognition (such as Google API) might be considered in the future. For the time being, the patient should be responsible for the activity choice, although this task increases the level of his/her engagement. The application then collects the location, weather condition (temperature, pressure and humidity) and the cardiovascular features that are added to the database.

However, the operation of such a system is highly dependent on user behavior and dedication: the duration of records, as already said, should be at least 300 seconds. An experiment with 10 healthy student-volunteers (Table 1) has shown that they rapidly lose the interest in data harvesting. The students were told to use the existing android application at least once a day, during one month. The students were healthy, without the need to check their status, so their initial enthusiasm did not persist.

This obvious decline of enthusiasm for participating in such an experiment was a motivation to diverge from the mere data harvesting and to include a sort of reward for the volunteers: each time they submit their estimated features, they get information about their current status, in respect to data of all the other volunteers recorded in similar environmental and ambient conditions. Such a feedback might inform them, for example, to reduce the level of the activities they are performing because their status is temporarily worsening.

There were proposals to include a discussion group within the hypertensive crowd sensing application, where subjects would discuss their status, feedback and mutually exchange the ideas and methods (crowdsourcing). This idea was, however, abandoned. Crowd sensing emphasizes its “sensing” component, i.e., minimal effort data collection (with feedback), while the chat on personal health diverts the application towards social groups. Besides, an application with a possibility to discuss the health issues at an unprofessional, non-medical, level might be non-ethical, or even against the law. Finally, advice that was beneficial to one patient might be disastrous for another one. These features might be left for the future, e.g., crowdsourcing groups. It would be particularly challenging to increase the level of patients’ motivation by giving them feedbacks according to the level of their participation. The adaptive feedback can be obtained using a scheme proposed in [21] (or similar), adopted to the particularities of the available cardiovascular data.

The privacy of the patient is another, closely related, issue. However, the patient does not transmit the complete signal that could be considered as a signature, but the signal features, i.e., signal reduced to 20–25 numbers. As soon as he gets a feedback, these number become nameless, just another set of statistical parameters included in the database. There is no trace if the patient sends the data every day or once in a life. The vulnerability of the patient and the feedback he gets is equivalent to the vulnerability of the operator. It should also be noted that the information transmitted to the patient is not permanent but related to the moment and a minute later his/her status might be considerably better (or worse). The privacy settings would also be improved using the bandwidth resources described in Section 2.3.1.

### 2.3. Things—Devices and Bandwidth

#### 2.3.1. Deployment of a TV White Space (TVWS) Network

IoE-enabled medical devices are expected to form a smart environment that is characterized by polymorphic requirements in terms of latency, throughput, reliability, speed, power, security, etc. generating enormous amounts of new, unstructured real-time data. Collecting, processing and validating data constitute the three most important operation steps of our proposed system. All three stages are associated with the success of running this system and must be considered in advance in order to maintain low cost, and high reliability as the number of users increases. In order for our crowdsourcing mechanism to be effective and statistically valid, a large number of ECG devices is required. These devices will generate data that will be fed into a dedicated network/cloud to be processed by a health-diagnostic engine. Connection of these devices is expected to be achieved through a wireless/cellular mobile network. Initially, existing established 2.5G/3G/4G networks can be employed to support the operation. This is provided that the data generated by the ECG is fed to the network through a mobile device or a dedicated transceiver operating on a cellular mobile network satisfying the minimum system requirements. Nevertheless, this could become an issue for users since the cost associated with constantly uploading sensor–data in real time over cellular networks can be high. Furthermore, existing cellular networks might experience high traffic (signaling) and increased bandwidth requests (data) that might not be able to support since the number of users and sensor devices is expected to increase over the years. Table 2 [22] lists a number of sensor types that could become part of our diagnostic engine.

Any significant increase in traffic will add to the complexity and cost of the network. To reduce traffic and support future healthcare applications, one can think of compressing the signals thus achieving a considerable reduction in the data that needs to be transmitted [23,24,25,26]. Nevertheless, some of these compression mechanisms might fail to preserve the clinical information in the processed data [26]. Another way to alleviate any future traffic demands is by introducing a low-cost wireless network, which can provide services to people in the cities as well as in rural areas where cellular coverage is limited.

For this, we propose the deployment of a TV White Space (TVWS) network that will operate with the support of a Geo-Location Spectrum Database (GLSD). Such a network can be deployed across a city as well as in rural areas. The amount of bandwidth allocated per user can vary depending on a number of factors, including the number of devices connected, and how many channels are available at any given time and at any given location. Although TVWS channels have a relatively small bandwidth (6, 7 or 8 MHz per channel) and channel availability is often of non-contiguous nature, optimal non-contiguous channel aggregation can be an attractive option to address these issues making full use of the available TVWS spectrum and achieve higher throughput if required [27]. As part of this communication system, a device—called Body Sensor Managing Device (BSMD) is introduced to locally (on the body) communicate with the ECG, while it transmits the data to the internet through unlicensed TV White Space (TVWS) spectrum employing the IEEE 802.22 [28].

Users carrying their BSMD equipment, are expected to move through the 802.22 cellular network and connect to the closest base-station to achieve internet access. The IEEE802.22 cellular network, to avoid causing/receiving interference to the incumbent devices, sensing-assisted spectrum databases (SASDs) can be used in conjunction with GLSD, eliminating the need for wireless network operations and management in a complex, interference-prone local or indoor environment [29]. Since healthcare has very strict operational requirements, a dynamic spectrum management will be employed relying on the information collected and managed by such databases. This will ensure incumbent protection, co-existence and interference management as well as fine-grained adaptation to available spectrum. The proposed TVWS network can be used in conjunction with the terrestrial networks to ensure that there is enough capacity in areas where high traffic is experienced.

#### 2.3.2. Hardware and Android application

**Figure 4:**
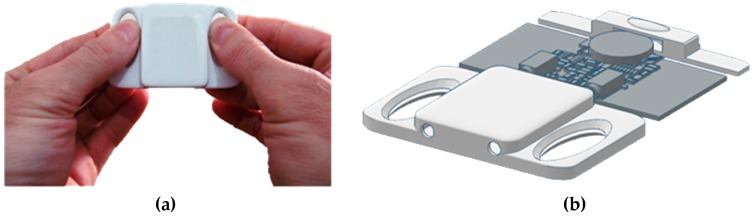
Mobile ECG sensing extender, usage and design by ECG for everybody: (**a**) its size compared with the fingers; (**b**) its interior with the plastic cover removed.

One of the preliminary hardware realizations is an additional sensing device [30], a concept that converts biomedical to audio signals for easy processing in smartphones. This concept implements the developed mobile ECG sensing extender (Figure 4) that enables capturing ECG with or without electrodes which makes this device flexible and easy to use in different situations and for different purposes.

**Figure 5:**
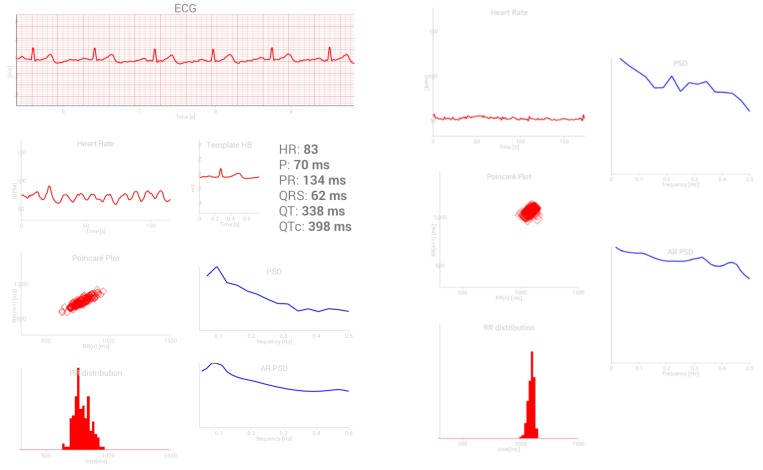
ECG for Everybody, an Android application available at [30]-a printout summary of a signal (lengths up to 120 s); the ECG signal is stored in database, while the participant, at his/her mobile device, can observe Poincaré plots.

Without an additional device, the smartphone camera [30] itself enables the heartbeat detection and further analysis (Figure 5). The current database contains above more than 150.000 self-annotated records of duration two minutes or less. The analytical results are visualized on the patient’s own mobile device (Figure 5) and serve as an additional motivation for the crowd sense activity participation, as similar visualization is not a part of a standard cardiovascular examination.

### 2.4. Process—Experimental Setting

#### 2.4.1. Signals and Features

In order to check both the reliability of the recorded data and the reliability of the “reward” the volunteers would get, we tested the signals recorded from 402 hypertensive patients. Some of the patients were undergoing therapy (consuming one of the following drugs: Alopres, Concor, Enalapril, Indapamide, Karvileks, Lisonorm, Lorista, Physiotense, Propranolol, Tenaxum, Verapamil) and some of them had not yet started the therapy or had refused to start therapy at all. As a control for machine learning, signals recorded from 128 healthy (examined) subjects were tested as well. The written permission is obtained from each subject. The typical cardiovascular features were extracted. These features are divided into nine groups as follows:

(1) Age
(2) Heart rate mean and standard deviation;
(3) Poincaré plots (PPlot or PP) of RR intervals parameters [31]:
  - standard deviation SD1 across the identity line of PPlot shows short-term variability,
  - standard deviation SD2 along the identity line of PPlot shows long-term variability,
  - ratio SD1/SD2;
  - copula parameter θ shows the level of interconnection of adjacent RR samples in PPlot plane [32];

PPlot features are long believed to be non-linear parameters, however, they can be expressed as a linear combination of the statistical moments [31].

(4) Detrended fluctuation analysis (DFA) is a method for determining the statistical self-affinity of a signal, with overall self-affinity α and its lower and upper segments α_1_ and α_2_ [33];
(5) Hurst exponent is similar to DFA but requires stationary data. It is used to explore the long-term memory of the time series [34];
(6) pNN50 is a percentage of adjacent pairs of NN intervals that differ more than 50ms. It is a tricky parameter as it must be performed on NN intervals (emphasized by its name: pNN50). NN intervals are RR intervals without artifacts and pathologies. Therefore, the signal must be pre-processed prior to applying this analysis;
(7) RMSSD is a root mean square of the adjacent NN intervals;
(8) ApEn, SampEn, and BinEn: Approximate entropy, ApEn, [35] is one of the most quoted methods for estimating the self-regularity of the observed process. Its improved modification is Sample entropy, SampEn, [36]. Some evaluation of their respective thresholds is specified in [37]. Both methods require artifact-free stationary data. Binarized entropy, BinEn, is more robust. It is derived for crowd sensing [17].
(9) Frequency parameters include the ratio of powers in low frequency and high frequency bands. The frequency bands range from 0.15 to 0.4 Hz–high (HF), from 0.04 to 0.15 Hz–low (LF), and from 0.0033 to 0.04 Hz–very low (VLF) [38].

The parameters are presented in Table 3. For the sake of comparison, the parameters are extracted both from the raw data (without the correction) and from the pre-processed (corrected) data. The algorithmic complexity for parameter evaluation is linear. The exceptions are entropy estimates ApEn and SampEn with quadratic complexity and for this reason BinEn is introduced [17]. BinEn is proven to be a low complexity entropy estimate. A detailed complexity analysis of each one of its algorithmic steps is given in [17]. It is shown that the impact of BinEn for patient classification exceeds the impact of ApEn and SampEn.

The analysis of cardiovascular parameters is a challenging task, as the most distinctive features, the systolic and diastolic blood pressure, are not available with sufficient reliability and accuracy in crowd sensing environment.

The parameters are evaluated for hypertensive raw data (HYP-R, HR), hypertensive corrected data (HYP-C, HC), control raw data (CNTRL-R, CR) and control corrected data (CNTRL-C, CC). Statistical significance between the groups is estimated pairwise using t-test for equal and unequal groups. The results are given in the four rightmost columns. Signals that exhibit a difference in the statistically significant way are denoted “1”.

A significant difference exists between the features extracted both from the raw and from the corrected data, except in a couple of cases that include the binarized entropy designed specifically for crowd sensing and therefore insensitive to artifacts. On the other hands, the artifacts did not alter the difference that exists between the features extracted from the patients and from the control, healthy group.

#### 2.4.2. The Central Application

Cloud computing [39] enables ubiquitous, convenient, on-demand network access to a shared pool of configurable computing resources (e.g., networks, servers, storage, applications, and services). These resources can be released with minimal management or service provider efforts and provided immediately to the customer. So for the central application that stores the features and provides the feedback to the patient we opted to use Platform as a Service (PaaS) in cloud [40].

The feedback to the patients contains the patient’s status, evaluated according to the features the patient transmitted. We opted to implement machine learning techniques (ML) for the feedback. ML is a branch of artificial intelligence that enables computers to learn from the sensors or databases, and to make decisions based on this knowledge. The goal is to model the complex non-linear relationship between the variables [41] within the groups of healthy control subjects and hypertensive patients. The model is applied to a binary classification problem. It predicts the affiliation to one of the classes, it defines the feature importance and, consequently, the feature selection.

Machine learning techniques have already been implemented in discrimination of cardiovascular parameters. One of the earliest smart-phone applications [42] performed the classification of heart beat shapes. A recent contribution [43] use the routine clinical data, including the blood pressure, to improve the cardiovascular risk prediction. In [44] and [45] the authors provide a review of the performance of several methods, with the application possibilities ranging from the cardiovascular disorders to the gene expression. To the best of authors’ knowledge, ML was not used to model the hypertension without the blood pressure as its key parameter, in moving patients with sensors attached without the supervision of medical staff.

We checked the appropriateness of two machine learning techniques: one was Artificial Neural Networks (ANN) and the other was Random Forest (RF). As one class of machine learning technique, Artificial Neural Networks (ANN) are efficient tools for classification and prediction. They are capable of modeling the complex non-linear relationship between a number of variables [46]. Their structure mimics the mechanism of transmitting neural signals following the genuine neural paths, in the process of making decisions about the outcome.

In this study, we used a Multi-Layer Perceptron (MLP) neural network with backpropagation [47]. It consists of computational units (neurons) with links labeled by different weighting coefficients, similar to neural axons, and with a sigmoidal transfer function. Process of learning means adjusting weights of connections through the minimizing errors on training data set.

Random forest [47] is an ensemble method. It consists of many building blocks, decision trees. Decision tree is another, simpler ML technique based on splitting the data into the subsets, as precise as possible. We used two different algorithms that implement decision tree for classification problems:

- ID3 (Iterative Dichotomizer 3) which uses Entropy function and Information gain as metrics [47]
- CART (Classification and Regression Trees) → uses Gini Index (Classification) as a metric [47]

A random forest algorithm generates many decision trees that model random sets of data and random sets of features. The decision is made by voting between all of the individual decisions. The input data set consists of parameters shown in the Table 3, evaluated from 402 hypertensive patients and 128 healthy control subjects. In contrast to RF, MLP strongly requires additional range normalization.

The data set is split into training (70%) and test (30%) set. Then the iterative procedure is applied: the training process yields a model fitted to the training data; the quality of the model is evaluated; if unsatisfactory, the training process restarts with different parameters. The number of iterations was enormous, yielding the final models with the properties considering accuracy, sensitivity, specificity, positive prediction and negative prediction shown in Table 4 [48].

**Figure 6:**
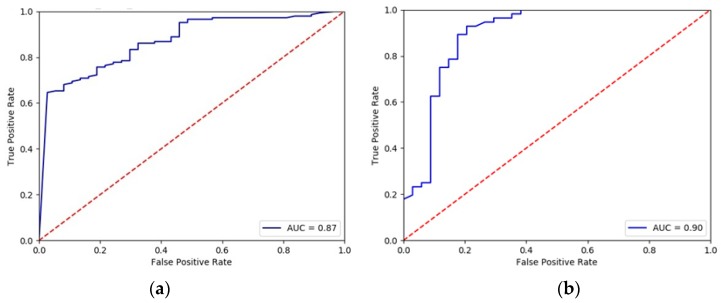
ROC curves for (**a**) Multi-Layer Perceptron and (**b**) Random Forest.

The Receiver Operating Characteristics (ROC) [48] curves are another tool for performance measurement. They show diagnostic abilities of a model at various thresholds settings. The area under the curve (AUC) shows how well the test separates the subjects into those with and without the hypertension. Figure 6 shows that both methods are ranked as “good”, i.e., with AUC within 0.8–0.9.

According to the results presented in Figure 5 and Figure 6, the random forest algorithm exhibited slightly better results, so this technique was chosen for the central application. With an established model, each feature set transmitted from the patients gets a probability to belong to the hypertensive group in the observed circumstances (sitting, running, working …).

**Figure 7:**
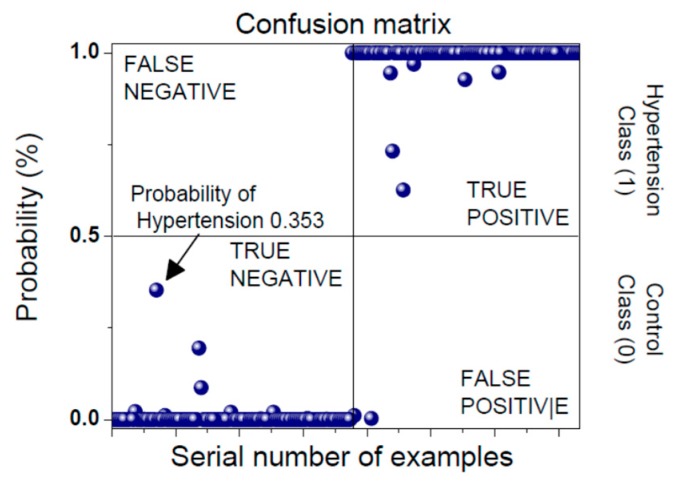
Confusion matrix: an arrow shows the decision considering the new feature set.

The classification results are presented in a form of a confusion matrix (Figure 7). Prediction classes are labeled as Negative (Healthy Control) and Positive (Hypertension). The model is built according to the training data set. A test set exhibits one of the four outcomes: True Negative, False Negative, True Positive and False Positive. An established model enables the evaluation of a Positive outcome probability for every incoming signal. An example in Figure 7 shows the probability that the input data belongs to a patient that is in a hypertensive state is equal to 0.353. This subject currently belongs to the healthy class, but with a high risk of entering a hypertension state, so he/she should decrease his/her activities.

**Figure 8:**
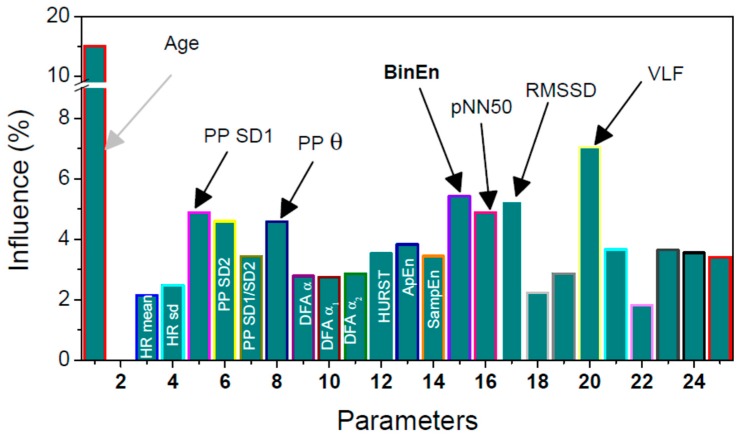
An influence of parameters considering the subject classification. The parameters with significant influence are labelled in black. Parameters 19–25 are related to the power spectral density. Parameter 18 is Total power (sum of the power spectral density components).

The feature importance was assessed using the mean decrease impurity method [46]. During the process of training, every node of every decision tree is a point of splitting, according to the importance of the single feature, measured by the chosen metric. This calculation implies the level of decreasing the weighted impurity in a tree for every feature. For a RF, the mean value of impurity decrease for a single feature is obtained by averaging values of impurity decrease for features in every tree. The feature importance is presented in the Figure 8. It is shown that the short term variability of the NN time series, determined by PPlot parameter SD1, is important for discriminating hypertensive from healthy patients. Difference between the adjacent NN intervals also reveals the short term variability, so the corresponding parameters are also of increased significance: pNN50, the percentage of successive NN intervals that differ more than 50 ms; RMSSD, the root mean square of differential NN time series; and BinEn, the entropy estimate of binary differentially coded NN time series. BinEn is developed for crowd sensing applications and for this reason it is bolded. As expected, the most significant feature is patient’s age.

## 3. Results

The previous analysis showed that the automatic pre-processing is sufficient to eliminate the artefacts from the extracted RR interval time series, implying the reliability of the data-base in cloud. The unoccupied spectral bandwidths could be used for transmission, and the transmission itself, as a major power consumer within the patient’s mobile device, would be reduced by sending carefully selected features.

Preliminary investigation has also shown that the awareness of the need for a large database of hypertension data in a variety of circumstances is not sufficient to motivate the patients to make the records of sufficient length, nor to persist in submitting them. For this reason, a feedback is provided, informing the patients about their current status. For this reason, two machine-learning techniques, random forest and multi-layer perceptron (MLP) neural network, were investigated, with the first one yielding the best one out of the investigated models. The feature importance was studied as well.

Although the hypertensive patients have distinctive diagnostic features–increased systolic and diastolic blood pressure values–their accurate and reliable values cannot be measured without the cuffs, which are semi-invasive, so not applicable for MCS. However, it was shown that parameters extracted from ECG are sufficient to make a distinction between the hypertensive and normal status of the patients with satisfactory accuracy.

## 4. Discussion

The purpose of this work is to collect the data (that would otherwise be deleted) from the hypertensive patients using their mobile wearable smart devices. The goal is to create a database with records created in various circumstances (work, walk, exercise, etc.) as a foundation for future research. The confronted challenges included the absence of the major distinctive parameter for hypertension, blood pressure, but also artifacts, battery and bandwidth (BBA) issues. The patients-volunteers are motivated to participate as they receive feedback: a model of the complex relationship of cardiovascular features is built, based on random forest algorithm, so the patients are informed about their current status considering the task they are performing. This crowd sensing system could be implemented, with slight modification, for any cardiovascular disease.

An extension of this concept will include GPS coordinates of each subject and automatic acquisition of the corresponding regional meteorology data (temperature, humidity) that affect the cardiovascular parameters. Besides the automatically collected data, the participants would respond to a questionnaire about their subjective feelings (well, headache, dizziness, insomnia); once logged in, the participants could get a feedback about general health attitude of the hypertensive neighbors. So the patients would be additionally motivated to join the crowd sensing, as they would be able correlate their subjective feeling and the environmental and weather conditions. Additional quality improvement would be to cooperate with another MCS system with complementary goal. An example is the *WiFiScout* system [49] which helps smartphone users find good quality WiFi hotspots, thus reducing the probability of erroneously received data. Future contribution would also include the development of parameters specifically for crowd sensing–robust, with low CPU consumption and artifact insensitive, just like BinEn [17]. Another challenging and important goal would be to develop a non-invasive but reliable blood-pressure sensor compatible with the crowd sensing environment.

## Figures and Tables

**Table 1:** Subjects and record lengths.

	No. of Subjects	Average Duration [s]
Day 1:	10	260 ± 52
Day 10:	3	120 ± 14

**Table 2:** Health Sensors and Bandwidth Requirements.

Sensor Type	Sample Rate	Bandwidth Consumed
ECG	125–500 sample/s.	2 kbps to 8 kbps based on 2 bytes per sample
Blood Pressure	1 sample/2 min	16 bits/1 min
Pulse	2 sample/s.	32 bps
Respiration	50 sample/s.	800 bps
SpO_2_	2 samples/s.	32 bps

**Table 3:** Values of the cardiovascular features of hypertensive (HYP) and control (CNTRL) subjects; the signals are raw (R) and corrected (C).

		CNTRL-R	CNTRL-C	HYP-R	HYP-C	CR vs CC	HR vs HC	CR vs HR	CC vs HC
HR mean	[bpm]	72.04 ± 0.96	71.92 ± 0.97	75.25 ± 0.95	74.94 ± 0.94	**0**	**0**	1	**0**
HR st. dev.	[bpm]	6.06 ± 0.25	4.67 ± 0.17	5.77 ± 0.29	3.86 ± 0.13	1	1	1	1
PP SD1	[ms]	43.87 ± 2.57	25.74 ± 1.38	36.72 ± 2.87	16.61 ± 0.86	1	1	1	1
PP SD2	[ms]	87.75 ± 3.37	73.18 ± 2.93	67.88 ± 2.47	56.28 ± 1.77	1	1	1	1
PP SD1/SD2		0.48 ± 0.02	0.35 ± 0.01	0.47 ± 0.02	0.29 ± 0.01	1	1	1	1
PP **q**		7.97 ± 0.35	8.12 ± 0.36	10.35 ± 0.40	11.04 ± 0.39	**0**	**0**	1	1
DFA **a**		0.85 ± 0.01	0.91 ± 0.01	0.90 ± 0.01	0.97 ± 0.01	1	1	1	1
DFA **a1**		0.80 ± 0.02	0.88 ± 0.02	0.90 ± 0.01	1.01 ± 0.01	1	1	1	1
DFA **a2**		0.81 ± 0.02	0.85 ± 0.02	0.84 ± 0.84	0.87 ± 0.02	**0**	**0**	**0**	**0**
HURST		0.77 ± 0.01	0.81 ± 0.01	0.80 ± 0.01	0.84 ± 0.01	1	1	1	1
SampEn		1.03 ± 0.03	1.15 ± 0.02	0.86 ± 0.02	0.99 ± 0.02	1	1	1	1
ApEn		1.04 ± 0.02	1.13 ± 0.02	0.90 ± 0.02	1.02 ± 0.01	1	1	1	1
BinEn		0.62 ± 0.01	0.62 ± 0.01	0.65 ± 0.00	0.65 ± 0.00	**0**	**0**	1	1
pNN50	[%]	18.01 ± 1.80	16.35 ± 1.66	8.38 ± 0.95	6.17 ± 0.75	**0**	1	1	1
RMSSD	[ms]	62.01 ± 3.63	36.38 ± 1.95	51.89 ± 4.05	23.48 ± 1.22	1	1	1	1
ULF%	[%]	98.28 ± 0.16	99.70 ± 0.02	94.00 ± 1.44	98.59 ± 0.39	1	1	1	1
VLF%	[%]	0.44 ± 0.04	0.10 ± 0.01	4.86 ± 1.40	1.32 ± 0.39	1	1	1	1
LF%	[%]	0.71 ± 0.07	0.14 ± 0.01	0.40 ± 0.05	0.06 ± 0.00	1	1	1	1
HF%	[%]	0.57 ± 0.08	0.07 ± 0.01	0.74 ± 0.13	0.03 ± 0.00	1	1	1	1
LF/HF		2.42 ± 0.22	2.98 ± 0.24	3.16 ± 0.27	3.94 ± 0.28	1	1	1	**0**
%LF/(LF+HF)	[%]	60.98 ± 1.66	66.13 ± 1.55	60.09 ± 1.67	69.92 ± 1.10	1	1	1	**0**
%HF/(LF+HF)	[%]	39.02 ± 1.66	33.87 ± 1.55	39.91 ± 1.67	30.08 ± 1.10	1	1	1	**0**

**Table 4:** Classification performance [%].

ML Techniques	Accuracy	Sensitivity	Specificity	Positive Prediction	Negative Prediction
MLP	85.6	87.3	82.9	88.9	80.0
RF	87.8	88.1	87.1	92.9	79.4
